# Early-Life Obesity Prevention: Critique of Intervention Trials During the First One Thousand Days

**DOI:** 10.1007/s13679-017-0255-x

**Published:** 2017-04-22

**Authors:** John J. Reilly, Anne Martin, Adrienne R. Hughes

**Affiliations:** 1 Physical Activity for Health Group, University of Strathclyde Glasgow, Glasgow, Scotland G1 1XQ UK; 2 Usher Institute for Population Health Sciences and Informatics, University of Edinburgh, Edinburgh, Scotland EH16 4UX UK

**Keywords:** Early-life interventions, Obesity prevention, Pediatric obesity, Infants, Children, Review

## Abstract

**Purpose of Review:**

To critique the evidence from recent and ongoing obesity prevention interventions in the first 1000 days in order to identify evidence gaps and weaknesses, and to make suggestions for more informative future intervention trials.

**Recent Findings:**

Completed and ongoing intervention trials have had fairly modest effects, have been limited largely to high-income countries, and have used relatively short-term interventions and outcomes. Comparison of the evidence from completed prevention trials with the evidence from systematic reviews of behavioral risk factors shows that some life-course stages have been neglected (pre-conception and toddlerhood), and that interventions have neglected to target some important behavioral risk factors (maternal smoking during pregnancy, infant and child sleep). Finally, while obesity prevention interventions aim to modify body composition, few intervention trials have used body composition measures as outcomes, and this has limited their sensitivity to detect intervention effects. The new WHO Healthy Lifestyles Trajectory (HeLTI) initiative should address some of these weaknesses.

**Summary:**

Future early obesity prevention trials should be much more ambitious. They should, ideally: extend their interventions over the first 1000 days; have longer-term (childhood) outcomes, and improved outcome measures (body composition measures in addition to proxies for body composition such as the BMI for age); have greater emphasis on maternal smoking and child sleep; be global.

## Introduction

**Fig. 1:**
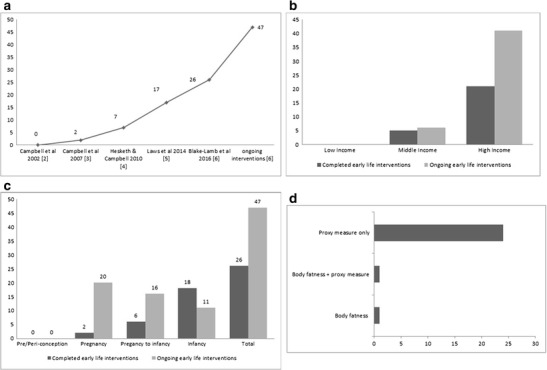
**a** Number of obesity prevention interventions in early life (first 1000 days) identified by successive systematic reviews. **b** Number of completed and ongoing early-life interventions by geographical region [2 ^•^]. **c** Number of early-life obesity prevention interventions by life-course stages [2 ^•^]. **d** Number of early-life obesity prevention studies reporting body composition (body fatness) outcomes [2 ^•^]

As the obesity pandemic has evolved, research efforts aimed at addressing it have increasingly focused on the opportunities for prevention which exist in early life. For the purposes of the present review early life is defined as pre or peri-conception, pregnancy, infancy, and early childhood (up to age 24 months), i.e., equivalent to the “first 1000 days” of life. This trend towards an emphasis on early-life obesity prevention was expressed in the recent Ending Childhood Obesity Report [1•] which highlighted the central importance of environmental modifications during pre-conception, pregnancy, infancy, and early childhood. Figure 1a demonstrates the rapidly increasing numbers of obesity prevention interventions in early life, as revealed by systematic reviews [2•, 3–6] published between 2002 and 2016.

Two fundamental arguments have been made for the increased focus on early-life preventive interventions. First, a number of common behavioral factors (including biological factors which are highly amenable to modification by behavior change, such as rapid infant growth) in early life substantially increase the risk of later obesity through establishing lifestyle habits which are obesogenic, and/or by “programming” of biological processes (such as appetite regulation) at critical times in development. There is now a substantial literature on early-life behavioral risk factors for childhood obesity, summarized and synthesized in recent systematic reviews. Monasta et al. [7] concluded that there was well-established evidence for the following risk factors: maternal diabetes; maternal smoking; rapid infant growth; no or limited breastfeeding; infant obesity; low physical activity in early childhood; short sleep duration in early childhood; and sugar-sweetened drink consumption in early childhood. More recently, Woo-Baidal et al. [8] concluded that the following early-life risk factors were very well-established: higher maternal pre-pregnancy weight status (e.g., body mass index—BMI); prenatal exposure to tobacco; excess maternal weight gain during pregnancy; higher infant birth weight; and rapid infant weight gain. Woo-Baidal et al. [8] also found that the following risk factors were fairly well-established: gestational diabetes; child care attendance; short sleep duration; introduction of complementary feeding before 4 months of age; and use of antibiotics in infancy. The second fundamental argument for preventive intervention in early life lies in the increasing concern over social inequalities in obesity—the generally higher risk of obesity with lower socio-economic (SES) in high-income countries [8, 9]. These social inequalities are already very marked by early childhood, and may be widening in high-income countries [10]. An emerging body of evidence suggests that targeting the well-established early-life behavioral risk factors for obesity (many of which are markedly socially patterned) may be particularly effective for reducing social inequalities in obesity [11•].

While early-life prevention of obesity is increasingly important to researchers [1•], it is a relatively new concept to many outside the pediatric obesity research community. There remains a good deal of uncertainty as to how best to turn the principles of early-life prevention into practical and effective strategies which can be incorporated into policy. The present review aims to critique the evidence base on early-life preventive interventions by providing evidence-based answers to the following research questions:

1. Where, geographically, have the early-life obesity prevention interventions taken place?;
2. Which life-course stages have research interventions focused on?;
3. Which of the well-established behavioral risk factors have research interventions targeted, and which have been neglected?;
4. What is the efficacy of early-life preventive interventions?;
5. To what extent is the evidence on efficacy of preventive interventions limited by study outcome measures?

## Methods

Since the present review aimed to critique the evidence on early-life childhood obesity prevention interventions, it was important that the critique was applied to a recent and comprehensive summary of relevant evidence. The 2016 systematic review by Blake-Lamb et al. [2•] on interventions for prevention of obesity in the first 1000 days of life was identified as a very good basis for the present critique. The Blake-Lamb et al. review [2•] addressed research questions which were distinct to those of the present critique, and was particularly useful in that it included ongoing (registered) trials as well as completed trials, so could provide some indication of future as well as past research efforts in this area. The relevance of the Blake-Lamb et al. review [2•] to the present critique was confirmed formally, using the relevance domain of the risk of bias in systematic reviews (ROBIS) method [12].

## Results

### Evidence-Based Answers to Our Research Questions

#### Question 1: Where, Geographically, Have Early-Life Obesity Prevention Interventions Taken Place?

While obesity is pandemic, and is placing a large and rapidly increasing burden on low-middle-income countries [1•, 10] the early-life obesity prevention intervention studies reviewed to date are far from global. Figure 1b illustrates the extent to which previous and ongoing trials have been biased towards high-income nations: of the 26 completed trials included in the review by Blake-Lamb et al. [2 ^•^] only 5 were from middle-income countries and none were from low-income countries, using the classification of national income status provided by the World Bank in 2016 (Blogs.worldbank.org/opendata/new-country-classifications2016); of the 47 ongoing trials identified by Blake-Lamb et al. [2•] only 6 were from middle-income countries and none were from low-income countries. Searching for studies published in English may have biased the search results to some degree against studies from low- and middle-income nations, but the lack of evidence from low- and middle-income countries is both stark and unfortunate.

#### Question 2: Which Life-Course Stages Have Been Targeted in Early-Life Obesity Prevention Interventions?

While early-life prevention of obesity encompasses any or all of the four potential life-course stages (pre-conception; pregnancy; infancy; early childhood, i.e., up to 24 months) considered by the present review, our critique shows that the evidence base to date is biased towards pregnancy and infancy (Fig. 1c **)**. Of the 26 completed trials identified by Blake-Lamb et al. [2•], no trials focused on pre-conception, 6 started during pregnancy and continued into infancy, 2 were pregnancy-only interventions, and 18 began their intervention during infancy. Blake-Lamb et al. [2•] identified a further 47 ongoing trials, and these provide a more current picture of the target populations in intervention studies. Among these 47 ongoing studies no trials targeted the pre-conception periods, 20 targeted pregnancy, 16 targeted pregnancy and infancy, and 11 focused on infancy (some of which extended interventions into early childhood).

In summary, published evidence is lacking from interventions which range across all four of the life-course stages, from interventions targeting pre- or peri-conception, and from interventions targeting the period 12–24 months of postnatal life. As noted above, multiple well-established and modifiable behavioral risk factors for obesity prevention exist during these “neglected” life-course stages (e.g., pre-pregnancy overweight and obesity; maternal smoking pre-pregnancy or during pregnancy; early childhood sleep, physical activity, and sugar-sweetened drink consumption).

#### Question 3: Which Behavioral Risk Factors Have Been Targeted in Early-Life Obesity Prevention Interventions, and Which Have Been Neglected?

Many well-established and modifiable early-life risk factors for obesity have been identified [7, 8]. In this section, we consider the extent to which the evidence of behavioral targets from preventive interventions maps onto the intervention evidence summarized by Blake-Lamb et al. [2•].

##### Risk Factors Targeted During Pre-conception Interventions

As noted above, none of the 73 trials identified by Blake-Lamb et al. [2•] focused on this stage of the life-course, and so no critique of the risk factors which were targeted was possible.

##### Risk Factors Targeted During Pregnancy

Among the 26 completed trials identified by Blake-Lamb et al. [2•], 2 focused on pregnancy only—neither targeted maternal smoking. Among the 47 ongoing trials identified, 20 focused on pregnancy (1 on managing gestational diabetes; 2 on fatty acid supplementation; 17 on maternal diet and/or physical activity). As with the completed trials, it appears that maternal smoking has been neglected as a potential intervention target, and a number of trials have targeted risk factors which are not particularly strongly supported by the evidence.

##### Risk Factors Targeted During Both Pregnancy and Infancy

Of the 6/26 completed trials which extended interventions across both pregnancy and infancy, 2 focused on targets not firmly established as risk factors for obesity (probiotics to alter infant gut microbiota; essential fatty acid supplementation); 2 focused on diet only (infant feeding); only 2 intervention trials targeted multiple behaviors (breastfeeding, complementary feeding, infant physical activity, family physical activity and diet in one trial; maternal diet and physical activity, infant and child feeding and physical activity, infant sedentary behavior and infant sleep in the other), not all of which were well-established as risk factors for childhood obesity. None of the trials appear to have targeted maternal smoking. The evidence base from completed pregnancy-infancy interventions is therefore quite limited, and does not map on to the evidence base on risk factors particularly well.

Among the ongoing trials during both pregnancy and infancy identified by Blake-Lamb et al. [2•], most (26 of 27) focused on pregnancy and infancy, while 1/27 extended the intervention from late infancy into early childhood: 2 used modified infant formula; 1 targeted breastfeeding promotion and another 1 targeted complementary feeding modification. The remaining 22 trials all involved multi-component interventions (diet, physical activity, only occasionally other risk factors such as sleep) targeting infants only (2 trials) or parents only (usually mothers, 8 trials) or both maternal and infant behaviors (12 trials). The behavioral targets of ongoing interventions in the pregnancy and/or infancy periods therefore map slightly better onto the risk factor evidence than the completed trials, but there remains a lack of emphasis on the targeting of maternal smoking, and a relative lack of trials focusing on the modification of infant sleep.

##### Risk Factors Targeted During Infancy and/or Early Childhood

Of the 18 completed trials of interventions which encompassed infancy only, early childhood only, or which crossed infancy-early childhood, 1/6 targeted infant sleep only, 1 targeted infant sleep combined with infant feeding; 4 targeted maternal/family diet and physical activity combined; 4 targeted infant feeding only, and 2 targeted infant feeding with physical activity and/or sedentary behavior; 4/18 attempted to modify infant growth via altering the composition (protein content or composition) of formula; 2/18 focused on less well-established potential biological risk factors (altering infant or maternal fatty acid intake). In summary, the extent to which the behavioral targets of these interventions mapped onto the behavioral risk factor evidence was quite limited. Specifically, in these 18 completed interventions there was limited targeting of sleep, and very limited targeting of physical activity, and sugar-sweetened drink consumption. There was also limited emphasis on the 12–24 month postnatal age.

Of the 11 ongoing trials in infancy, early childhood (12–24 months), or which crossed infancy and early childhood, 6 were in infants only: 2 involved modifications to infant formula; 2 targeted breastfeeding and complementary feeding; 2 were multi-component (infant feeding, physical activity, sedentary behavior and sleep). A single trial focused on the 12–24 month period only, and it targeted diet, physical activity, and sleep. All 4 of the trials which crossed both infancy and early childhood were multi-component, targeting infant feeding and infant/child/family diet, and physical activity; one trial also targeted sleep. The ongoing trials seem to have targeted a higher proportion of the behavioral risk factors than the completed trials, though sleep was under-represented as a target.

#### Question 4: What is the Efficacy of Early-Life Preventive Interventions to Date?

Future policy interventions would benefit from clear and consistent evidence of efficacy (e.g. from randomized controlled trials—RCT), effectiveness (e.g., application of the intervention beyond RCT, and scale-up), and cost-effectiveness of interventions. Of the 26 completed trials included in the review by Blake-Lamb et al. [2•] evidence of at least some efficacy, in the form of a statistically significant benefit to the intervention, was found in 11 studies. There may well have been publication bias, favoring the publication of trials with evidence of efficacy, though a strong bias in the opposite direction arises from the choice of outcome measures, discussed below. Many of the trials were limited: relatively short-term interventions in many cases; small (some were pilot studies); most had relatively short-term follow-up so could not provide evidence on effects into childhood; there was evidence of attenuation of intervention effects over time [e.g., 13]. Even when efficacy was established, for multi-component interventions (i.e., those which targeted more than one risk factor or behavior) it is difficult to attribute efficacy to the different components with any confidence.

In general, intervention effects seemed promising, but were of uncertain longer-term significance (given possible attenuation of intervention effects), and public health significance. For example, Navarro et al. [14] had a mean beneficial effect on child BMI *z*-score of −0.31 (CI 0.12 to 0.49) at 15–24 months; Wen et al. [15] found a lower mean child BMI of 0.29 kg/m^2^ (CI 0.02 to 0.55) at 24 months; Daniels et al. [13] found a mean intervention effect of 0.19 on BMI *z*-score at 13–15 months of age which had attenuated and was non-significant at 24 months of age. Taken together, the evidence to date suggests that efficacious interventions generally have modest effects on BMI. Achieving more substantial and longer-term effects might require interventions across several/all of the life-course stages considered by the present critique, so that by the achievement of multiple but modest additive effects the cumulative/overall effects are increased. The major exception to this is the trial of modification of the protein content of infant formula by Koletzsko and colleagues [16]. It hypothesized to reduce the rate of infant growth by reducing the protein content of formula so that it more closely resembled that of breast milk [16]. This trial was notable for providing evidence of both long-term effects on obesity as an outcome, and for demonstrating effects which were more substantial than the other trials: increased risk of obesity at age 6 years in the higher protein formula group (adjusted odds ratio 2.43, CI 1.12 to 5.27) [16].

#### Question 5: To What Extent is the Evidence on Efficacy Likely to Have Been Limited by Choice of Outcome Measure?

The 26 completed trials reviewed by Blake-Lamb et al. [2•] aimed to modify body composition (body fatness) favorably, but few measured body composition as an outcome as illustrated by Fig. 1d **.** One study had a measure of body fatness as an outcome, 1 had a measure of body fatness and a proxy for body fatness (BMI *z*-score), and the other 24 used proxies for body fatness (BMI *z*-score in most cases, or weight for height or length in a few, or single or multiple skinfolds in a few studies). There is substantial evidence that more direct measures of body composition, rather than crude proxies for body composition, are more likely to identify effects in pediatric clinical intervention trials [17]. Our recent longitudinal studies on the etiology of childhood obesity have been able to identify associations with exposures, such as sedentary behavior, when we have used measures of changes in body fatness as outcomes, but not when we have used proxies for change in body fatness (e.g., change in BMI or BMI *z*-score) in the same analyses [18]. Finally, our systematic review on physical activity in the etiology of childhood obesity found that associations between physical activity-adiposity (and effects of physical activity on adiposity in intervention studies) were much more likely to be identified when measures of body composition were used as outcome measures, rather than crude proxies for body composition such as BMI [19].

It should also be noted that the advantages of having a measure of body composition or change in body composition may not require especially sophisticated measures; even relatively simple field measures such as bio-electrical impedance can bring substantial benefit over proxy measures [18]. At present, the “criterion” measures of body composition with high accuracy are the multi-component methods. They require combined measurement of body density and total body water for a “three component” model measure of body composition and measurement of body density, total body water, and total body mineral for a “four component” model measure [20]. Multi-component measures are not practical for large field studies in obesity prevention. However, total body water measurement with stable isotope dilution is a highly accurate “reference method” [20, 21], with negligible bias. It is also much more suitable for field use than the criterion methods, and with an increasing body of evidence on its successful use in field studies in low-middle-income countries [21].

The above critique of BMI-for-age as an outcome measure does not negate its value as a simple obesity screening tool for clinical use, and for population surveillance of obesity. Systematic reviews have established that it has high specificity for identifying the fattest children and adolescents, and those with cardio-metabolic risk factors and co-morbidities, though with only moderate sensitivity [22–24]. However, future interventions would benefit from the use of body composition measures as outcomes (in addition to proxies such as BMI *z*-score).

## Discussion

The present critique was based on a very recent and highly relevant systematic review of interventions to prevent obesity in the first 1000 days [2•], and so our research questions have answers which are both current and highly evidence-based. The main findings of our critique are the quantification of a number of substantial gaps in the evidence on interventions for obesity prevention during the “first 1000 days”, and the identification of mismatches between the behavioral risk factor evidence and the intervention evidence. In particular, there was a clear lack of evidence from low- and middle-income countries, from pre-conception interventions, from interventions which extended over all four important stages of the life-course, from interventions in the period 12–24 months, from interventions which target some of the well-established risk factors (notably maternal smoking during pregnancy), a relative lack of interventions targeting some other well-established risk factors (e.g., sleep during infancy and childhood), and a lack of interventions with body composition measures as outcomes. Future research might usefully focus on these major gaps in the evidence.

Addressing the gaps and mismatches in the evidence identified by the present critique is not straightforward, and would require funding for intervention trials which is larger and more long-term than is usually available. For example, conducting the necessary intervention study which: runs from pre-conception to age 2 years, matches the intervention targets to the well-established behavioral risk factors, has higher quality and much longer-term outcome measures, (and extends—ideally—from efficacy to effectiveness and cost-effectiveness), would clearly be challenging in terms of the cost of the work/the long-term nature of funding required. Nonetheless, the current and future global burden of childhood obesity warrants an effort of this kind [1•, 25].

A major new research project coordinated by WHO, starting in 2017, the Healthy Lifestyle Trajectories Initiative, HeLTI (http://www.cihr-irsc.gc.ca/e/49510.html) could help fill many of the gaps in the intervention evidence base identified by the present critique. The HeLTI will use intervention cohorts and aims to (i) target interventions on the most important behavioral risk factors, (ii) extend interventions over many of the early life-course stages covered by the present review, (iii) base the cohorts in low-middle-income countries. The HeLTI, and future studies along similar lines, should be able to test the hypothesis suggested by the present critique that achieving a substantial impact on obesity prevention in early life will require modifications to multiple behavioral risk factors, extending over multiple life-course stages.

Future targeting of well-established behavioral risk factors, which have been identified as neglected by the present critique, will not be straightforward. For example, there is a substantial literature on interventions to help women stop/reduce smoking during pregnancy. This is a behavioral change which has proved difficult to achieve, but was achievable [e.g., 26]. A further difficulty is that families and healthcare practitioners, at least in high-income western countries, commonly have strongly held views about the early behavioral risk factors for obesity which are at odds with the evidence. For example, mothers and health visitors (community nurses with responsibility for the health of mothers, infants, and toddlers) in one English study reported the following strongly held views: infancy was “too early” to intervene for obesity prevention, and there was considerable potential for harm in doing so; infant crying was always a signal of hunger; and babies “cannot be overfed” [27]. Clearly, research and policy interventions must address such strong cultural barriers to early obesity prevention. Stressing the minimal potential for harm and the substantial potential benefit of targeting the behavioral risk factors in the first 1000 days may be helpful. In advocacy for the required larger and longer-term funding, for policy change, or for addressing cultural barriers to change, it may be worth reiterating the low risk and many important co-benefits to the main behavioral changes required for obesity prevention: maternal smoking cessation/reduction; adequate maternal physical activity and healthy body weight and composition pre-conception; healthy weight gain during pregnancy; appropriate breastfeeding and complementary feeding; improved sleep in infancy and early childhood; reductions in sugar-sweetened drink consumption in early childhood; promotion of infant and early childhood physical activity. All of these changes should bring substantial and long-lasting benefits to maternal and child health and well-being. Finally, theory and evidence-based behavioral interventions to improve the health of adolescents in low-middle-income countries are now becoming available [e.g., 28], and these have the potential to deal with the evidence gap from such countries, and the gap relating to the pre-conception stage of the life course highlighted by the present critique.

## Conclusions

Future research interventions aiming to prevent obesity in early life should, where possible, be more global with a much greater emphasis on populations in low-middle-income countries where childhood obesity is now prevalent, be careful to map intervention targets on to the behavioral risk factor evidence, be longer-term with both longer-term interventions (ideally extending over much or all of the first 1000 days, gaining from cumulative/additive intervention effects) and longer-term outcomes (extending into childhood) and use body composition measures as outcomes in addition to simple proxies for body composition such as BMI for age.
